# SPC25 as a novel therapeutic and prognostic biomarker and its association with glycolysis, ferroptosis and ceRNA in lung adenocarcinoma

**DOI:** 10.18632/aging.205418

**Published:** 2024-01-11

**Authors:** Xu-Sheng Liu, Yu Zhang, Xing Ming, Jian Hu, Xuan-Long Chen, Ya-Lan Wang, Yao-Hua Zhang, Yan Gao, Zhi-Jun Pei

**Affiliations:** 1 Department of Nuclear Medicine, Hubei Provincial Clinical Research Center for Precision Diagnosis and Treatment of Liver Cancer, Taihe Hospital, Hubei University of Medicine, Shiyan 442000, China; 2 Hubei Provincial Clinical Research Center for Umbilical Cord Blood Hematopoietic Stem Cells, Taihe Hospital, Hubei University of Medicine, Shiyan 442000, China; 3 Department of Infection Control, Taihe Hospital, Hubei University of Medicine, Shiyan 442000, China; 4 Department of Critical Care Medicine, Danjiangkou First Hospital, Danjiangkou 420381, China; 5 Department of Medical Ultrasound, Taihe Hospital, Hubei University of Medicine, Shiyan 442000, China

**Keywords:** SPC25, lung adenocarcinoma, glycolysis, cell cycle, ferroptosis, ceRNA

## Abstract

Objective: Spindle pole body component 25 (SPC25) is an important cyclin involved in chromosome segregation and spindle dynamics regulation during mitosis. However, the role of SPC25 in lung adenocarcinoma (LAUD) is unclear.

Materials and Methods: The differential expression of SPC25 in tumor samples and normal samples was analyzed using TIMER, TCGA, GEO databases, and the correlation between its expression and clinicopathological features and prognosis in LUAD patients. Biological pathways that may be enriched by SPC25 were analyzed using GSEA. *In vitro* cell experiments were used to evaluate the effect of knocking down SPC25 expression on LUAD cells. Correlation analysis and differential analysis were used to assess the association of SPC25 expression with genes related to cell cycle, glycolysis, and ferroptosis. A ceRNA network involving SPC25 was constructed using multiple database analyses.

Results: SPC25 was highly expressed in LUAD, and its expression level could guide staging and predict prognosis. GSEA found that high expression of SPC25 involved multiple cell cycles and glycolytic pathways. Knocking down SPC25 expression significantly affected the proliferation, migration and apoptosis of LUAD cells. Abnormal SPC25 expression levels can affect cell cycle progression, glycolytic ability and ferroptosis regulation. A ceRNA network containing SPC25, SNHG15/hsa-miR-451a/SPC25, was successfully predicted and constructed.

Conclusions: Our findings reveal the association of up-regulation of SPC25 in LUAD and its expression with clinical features, prognosis prediction, proliferation migration, cell cycle, glycolysis, ferroptosis, and ceRNA networks. Our results indicate that SPC25 can be used as a biomarker in LUAD therapy and a target for therapeutic intervention.

## INTRODUCTION

Lung cancer is a type of cancer that affects the lungs and is one of the leading causes of cancer-related deaths globally [1, 2]. Among various types of lung cancer, lung adenocarcinoma (LUAD) is the most common form, accounting for approximately 40% of all cancer cases [3]. However, despite advances in diagnostic capabilities, the prognosis for cancer remains poor. The overall survival rate for cancer patients is relatively low, primarily due to late diagnosis, rapid tumor growth, and limited treatment options. In recent years, research has found that cancer can be redefined as a metabolic disease [4], including LUAD [5]. The inherent biological invasiveness and metabolic reprogramming of LUAD contribute to its high rates of recurrence and progression [5–7]. Therefore, there is an urgent need to better understand the underlying mechanisms of cancer development and progression, and to identify new diagnostic biomarkers and therapeutic targets to improve patient management.

NDC80, a tetrameric protein complex composed of NDC80, NUF2, spindle pole body component 25 (SPC25) and SPC24, is crucial to the separation of chromosomes and the average distribution of them into daughter cells during cell division [8, 9]. The wrong allocation of chromosomes during cell division can cause birth defects, cancer, and other diseases, among which SPC25 has been found to be upregulated and increase cancer stem cell properties in lung cancer [10, 11]. Whereas, there are few studies concentrated on the relationship of SPC25 with tumor cell proliferation and energy metabolism in LUAD.

The core nature hallmarks of tumor cells are unlimited replication potential and avoidance of apoptosis [6]. Besides, metabolic reprogramming, which promotes the growth and metastasis of cancer cells, is emerging as a new cancer marker [12, 13]. Among them, the Warburg effect is an important discovery in metabolic reprogramming. The Warburg effect refers to the phenomenon in which cancer cells rely on glycolysis to produce energy under hypoxic conditions instead of relying on oxidative phosphorylation, which is a highly efficient metabolic pathway. This phenomenon is widely observed in tumor cells and is believed to be one of the important factors contributing to the rapid proliferation of tumor cells [14]. Research has also identified that ferroptosis [15–18] and ceRNA networks [19, 20] play pivotal roles in the occurrence and progression of cancer. Therefore, further investigation of the effect of SPC25 on LUAD, particularly through modulating tumor cell glycolysis, ferroptosis, and ceRNA biological functions, plays a crucial role in improving diagnosis and treatment of LUAD patients.

The aim of this study was to utilize bioinformatics analysis and *in vitro* experiments to investigate the expression differences of SPC25 in LUAD and its correlation with clinical pathological features and prognosis. Additionally, the interference of SPC25 expression was validated to significantly inhibit the proliferation and migration of LUAD cells. Furthermore, the potential molecular mechanisms in which SPC25 may be involved, as well as its relationship with glycolysis, ferroptosis and ceRNA network, were further explored. Our study will further advance the understanding of the mechanisms underlying cancer development and provide new insights and approaches for early detection, diagnosis, and treatment of tumors.

## MATERIALS AND METHODS

### SPC25 expression levels in cancers and prognostic value in LUAD, as well as association with clinicopathological characteristics for LUAD patients

We evaluated the expression of SPC25 in pan-cancer using the TIMER (https://cistrome.shinyapps.io/timer/) online tool [21]. In this study, we focused on its expression changes in the LUAD dataset. Additionally, we downloaded three LUAD datasets (GSE116959, GSE31210, and GSE7670) from the GEO database (http://www.ncbi.nlm.nih.gov/geo/) [22] to validate the differentially expressed of SPC25 between LUAD samples and normal samples. GEPIA online tool (http://gepia.cancer-pku.cn/) [23] was used to estimate the relationship of SPC25 levels with OS and DFS for LUAD patients, which log-rank *p*-values and HR were obtained simultaneously. Finally, we obtained the LUAD dataset from the TCGA database (https://tcga-data.nci.nih.gov/tcga/) [24] for subsequent analysis of gene expression correlations, gene expression differences, and clinical feature correlations. The glycolysis [25, 26] and ferroptosis [15, 16] related gene lists used in this study were referenced from previous research.

### Bioinformatic functional analyses of SPC25 in LUAD

To evaluate the potential involvement of the SPC25 gene in the biological pathways of LUAD, we performed GSEA analysis [27]. Initially, the TCGA LUAD dataset was divided into two groups, based on the expression levels of the SPC25 gene. The DESeq2 package [28] was utilized to identify differentially expressed genes between the high and low groups. Subsequently, the clusterProfiler package [29] was employed to perform GSEA analysis on the differentially expressed SPC25 genes in the TCGA LUAD dataset. The reference genes used were h.all.v2022.1.Hs.symbols.gmt (Hallmarks) and c2.cp.all.v2022.1.Hs.symbols.gmt (All Canonical Pathways). Data with FDR (*q*-value) < 0.25 and *p*.adjust < 0.05 were considered statistically significant. The gene set database used was the MSigDB collection (https://www.gsea-msigdb.org/gsea/msigdb/collections.jsp#C2).

### Cell culture and siRNA transfection

The human LUAD cell line (H1975) was purchased from BeNa Culture Collection (BNCC340345, Beijing, China). And it was cultured in Roswell Park Memorial Institute (RPMI) 1640 complete medium (KGM31800S) and incomplete medium (KGM31800N) from KeyGEN BioTECH (Jiangsu, China).

Five short interfering RNAs (siRNAs) targeting SPC25 (si-SPC25) and a scrambled control siRNA were designed and commercially synthesized by General Biosystems (Anhui, China). The target sequence is shown in Table 1. Based on the verification results, we ultimately chose si-216 and si-711 to continue with the subsequent experiments. Plant cells in different culture dishes according to experimental requirements. Once their density reached 70–80%, the siRNA was transfected into cells using Lipofectamine 3000 transfection reagent (L3000015, Invitrogen). For a detailed experimental protocol, please refer to our previous studies [30].

**Table 1:** The sequences of siRNAs used in our study.

**Gene**	**Sense**	**Antisense**
**SPC25 (human) siRNA-216**	GGACUAAGAGAUACCUACATT	UGUAGGUAUCUCUUAGUCCTT
**SPC25 (human) siRNA-576**	GGUGAGAAAUUGCAGUUUATT	UAAACUGCAAUUUCUCACCTT
**SPC25 (human) siRNA-711**	GAAUUUCAAGAGAAUGUAATT	UUACAUUCUCUUGAAAUUCTT
**SPC25 (human) siRNA-262**	AGCUGUCUGUGAAAUUAAATT	UUUAAUUUCACAGACAGCUTT
**SPC25 (human) siRNA-437**	GGAUCUUAAGGAAGAAUAUTT	AUAUUCUUCCUUAAGAUCCTT
**NC**	UUCUCCGAACGUGUCACGUTT	ACGUGACACGUUCGGAGAATT

### RNA isolation and quantitative real-time PCR assays

Total RNA was extracted using TRIzol Reagent (CW0580S, CWBIO). Quantitative real-time PCR was performed using the CFX Connect™ Real-time PCR system (CFX Connect™, Bio-Rad). The 2^−ΔΔCt^ method was used for quantitative analysis. We used β-actin as an internal control for normalization. Supplementary Table 1 contains the sequences for all primers.

### Western blotting

H1975 cells were treated with RIPA lysis buffer and protein concentration was measured using the BCA protein assay kit (E-BC-K318-M, Elabscience). After extraction, proteins were separated on SDS-PAGE gels and transferred to PVDF membranes (Millipore, Massachusetts, USA). The 5% non-fat milk were used to block above membranes, and we incubated them with the Anti-SPC25 antibody produced in rabbit (1:1000, DF13747, Affinity Biosciences) or Mouse anti-β-actin (1:2000, HC201, TransGen Biotech) at 4°C overnight. The membranes were washed with TBST, then incubated with corresponding secondary antibodies for 1 h at room temperature, namely HRP conjugated Goat Anti-Rabbit IgG (H+L) (1:2000, GB23303, Servicebio) and HRP conjugated Goat Anti-Mouse IgG (H+L) (1:2000, GB23301, Servicebio). Protein levels were detected using an enhanced chemiluminescence (ECL) system.

### Cell counting Kit-8 (CCK-8) and EdU proliferation assays

Cell viability was evaluated using the CCK-8 kit (KGA317, KeyGen) at 24, 48, and 72 hours of cultivation, respectively.

We used the Beyotime Biotechnology EdU kit to stain cells (Shanghai, China) according to the instruction. As regards result acquisition, we used a fluorescent microscope to capture images from five random fields. ImageJ was used to compare EdU positive (red fluorescence) cells with DAPI stained (blue fluorescence) cells.

### Wound-Healing

When the transfected cells reached 90% confluency, we scratched the cells using the tip of a 200 μL pipette. At the time of wound generation 0 h, 24 h, and 48 h, a microscope was used to observe the scratch. The gap width was measured using ImageJ.

### Cell cycle analysis and cell apoptosis assays

Cell Cycle Staining Kit (CCS102, MULTI SCIENCES) was used for cell cycle analysis and the Annexin V-FITC/PI Cell Apoptosis Kit (AP101-100-kit, MULTI SCIENCES) was used for apoptosis assays. The gene list of cyclins, cyclin-dependent kinases (CDKs), and cyclin-dependent kinase inhibitors (CDKIs) is referenced from previous studies [31–33].

### Lactate generation

According to the manufacturer’s instructions, the lactate colorimetric method was employed to determine the lactate levels in the culture medium. Specifically, the reagent kit (E-BC-K044-M) provided by Elabscience company (Wuhan, China) was utilized. To ensure accurate measurement of lactate levels, we followed the manufacturer’s instructions and measured the absorbance data at a wavelength of 530 nm.

### 2-NBDG uptake assay

H1975 cells were seeded in a 96-well plate at a density of 2 × 10^4^ cells per well. Subsequently, the cells were transfected with siRNA and washed with PBS within 24 hours post-transfection. Next, the cells were incubated in glucose-free DMEM supplemented with 50 μM 2-NBDG (HY-116215, MCE) at 37°C with 5% CO2 for 30 minutes. After the incubation period, the cells were washed with warm PBS, repeating this washing step for a total of three times. Finally, the average fluorescence intensity (MFI) of 2-NBDG was quantitatively measured using NovoCyte™ flow cytometry.

### CeRNA network prediction and analysis

In this study, we employed the microT-CDS (https://dianalab.e-ce.uth.gr/html/dianauniverse/index.php?r=microT_CDS) [34] and mirDIP (http://ophid.utoronto.ca/mirDIP/index.jsp#r, The Score Class is designated as Very High.) [35, 36] to predict target miRNAs directed towards SPC25, and validated the final target miRNAs through differential expression analysis. Additionally, we utilized the ENCORI (accessible at https://rnasysu.com/encori/index.php) [37] and miRNet (accessible at https://www.mirnet.ca/miRNet/home.xhtml) [38] platforms to predict lncRNAs targeted by the identified miRNAs, and identified the final target lncRNAs through differential expression analysis. By following the ceRNA hypothesis, we constructed the corresponding ceRNA network. To predict potential binding sites for mRNA-miRNA and lncRNA-miRNA interactions, we employed the RNAHybrid online tool (https://bibiserv.cebitec.uni-bielefeld.de/rnahybrid) [39].

### Statistical analyses

We performed comprehensive statistical analysis and graphical presentations using Xiantao online tool (https://www.xiantaozi.com/) and GraphPad Prism statistical software. Unpaired student’s *t*-test and the Wilcoxon test were employed to analyze the difference comparisons of three or more groups. The survival time was evaluated using Cox regression analysis and Kaplan Meier (KM) method. ^*^P-value < 0.05 was considered as statistically significance.

### Availability of data and materials

The datasets generated during and/or analysed during the current study are available from the corresponding author on reasonable request.

## RESULTS

### SPC25 expression levels in cancers and prognostic value in LUAD, as well as association with clinicopathological characteristics for LUAD patients

**Figure 1:**
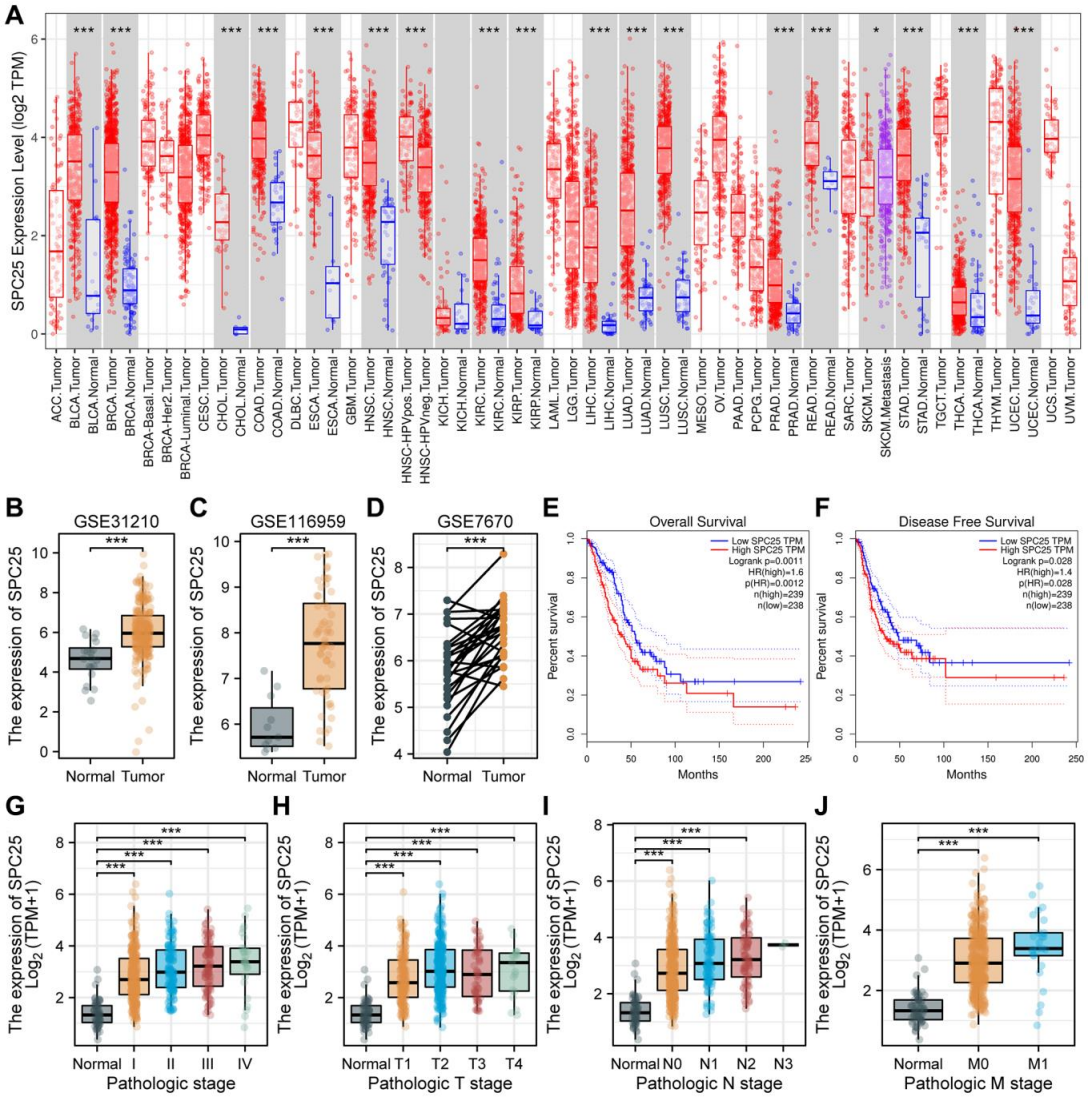
**SPC25 expression levels in cancers and prognostic value in LUAD, as well as association with clinicopathological characteristics for LUAD patients.** (**A**) SPC25 levels in different tumor tissues and paracancerous tissues based on the TIMER database. (**B**–**D**) SPC25 levels in LUAD samples and normal samples in the (**B**) GSE31210, (**C**) GSE116959 and (**D**) GSE7670. (**E**, **F**) Prognostic analysis of SPC25 expression on OS (**E**) and DFS (**F**) in LUAD based on the GEPIA database (log-rank test). (**G**) SPC25 expression was explored in different pathological stages based on the TCGA LUAD database. (**H**) SPC25 expression was explored in different pathological T stages based on the TCGA LUAD database. (**I**) SPC25 expression was explored in different pathological N stages based on the TCGA LUAD database. (**J**) SPC25 expression was explored in different pathological M stages based on the TCGA LUAD database. Abbreviations: TCGA: the Cancer Genome Atlas; LUAD: lung adenocarcinoma; OS: overall survival; DFS: disease free survival. (^***^p < 0.001; ^**^p < 0.01; ^*^p < 0.05; Abbreviation: ns: not significant).

In order to preliminarily understand the expression pattern of SPC25 in human cancer, we used the TIMER online tool for analysis. The results showed that compared with normal tissues, SPC25 expression levels showed obvious elevations in bladder urothelial carcinoma (BLCA), breast invasive carcinoma (BRCA), cholangiocarcinoma (CHOL), colon adenocarcinoma (COAD), esophageal carcinoma (ESCA), head and neck squamous cell carcinoma (HNSC), kidney renal clear cell carcinoma (KIRC), kidney renal papillary cell carcinoma (KIRP), liver hepatocellular carcinoma (LIHC), lung adenocarcinoma (LUAD), lung squamous cell carcinoma (LUSC), prostate adenocarcinoma (PRAD), rectum adenocarcinoma (READ), stomach adenocarcinoma (STAD), thyroid carcinoma (THCA) and uterine corpus endometrial carcinoma (UCEC) (*P* < 0.05, Figure 1A). Furthermore, for verifying SPC25 expression change in LUAD tissues, we then examined its level based on the above GEO datasets mentioned above (*P* < 0.05, Figure 1B–1D). Taken together, SPC25 level was generally increased in LUAD patients.

Survival analysis results obtained from the GEPIA database indicate that SPC25 was an important indication of overall survival (OS), disease free survival (DFS) in LUAD. There were significant survival time differences between SPC25 high- and low-expression subgroups. Patient outcomes clearly improved in those with lower expression of SPC25 (*P* < 0.05, Figure 1E, 1F). Collectively, these findings demonstrate that SPC25 may represent a valuable prognostic biomarker for LUAD.

As part of our investigation in the clinical significance of SPC25 in LUAD, we examined the relationship between SPC25 levels and multiple important clinicopathologic features in the TCGA-LUAD database. Through our analysis, it was found that the expression level of SPC25 in normal samples was significantly lower compared to stage I, II, III, and IV patients in the pathological staging analysis (*P* < 0.05, Figure 1G). In the pathological T staging analysis, the expression level of SPC25 in normal samples was significantly lower compared to T1, T2, T3, and T4 stage patients (*P* < 0.05, Figure 1H). In the pathological N staging analysis, the expression level of SPC25 in normal samples was significantly lower compared to N0, N1, and N2 stage patients, while statistical analysis could not be performed for N3 stage patients due to sample size less than three (*P* < 0.05, Figure 1I). In the pathological M staging analysis, the expression level of SPC25 in normal samples was significantly lower compared to M0 and M1 stage patients (*P* < 0.05, Figure 1J). Overall, our research results revealed the expression changes of SPC25 in different pathological stages, providing a strong foundation for further investigation into the biological function and clinical application of SPC25.

### Functional enrichment analysis of SPC25 in LUAD

**Figure 2:**
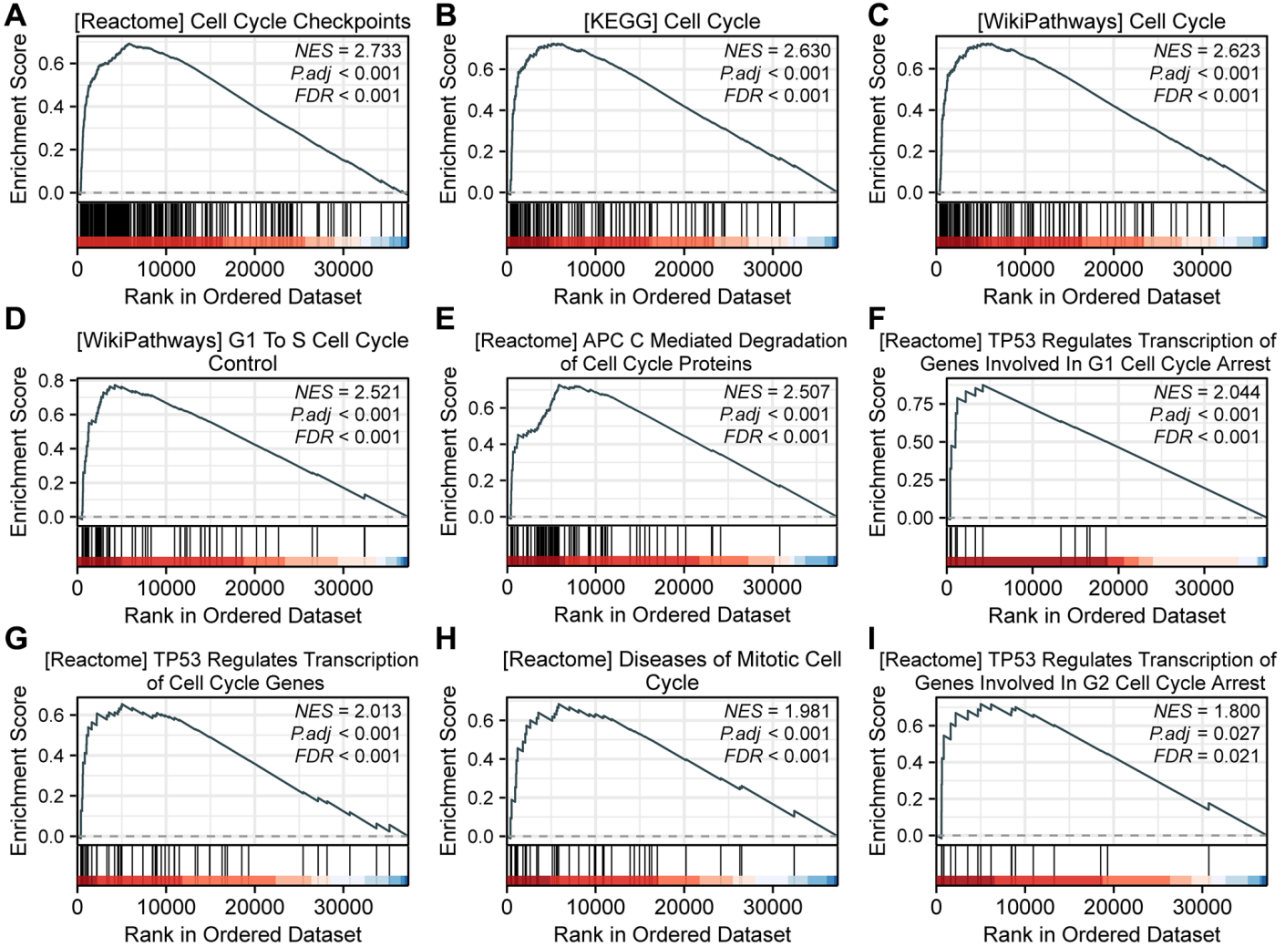
**GSEA analysis reveals SPC25-related pathways.** (**A**) REACTOME_CELL_CYCLE_CHECKPOINTS. (**B**) KEGG_CELL_CYCLE. (**C**) WP_CELL_CYCLE. (**D**) WP_G1_TO_S_CELL_CYCLE_CONTROL. (**E**) REACTOME_APC_C_MEDIATED_DEGRADATION_OF_CELL_CYCLE_PROTEINS. (**F**) REACTOME_TP53_REGULATES_TRANSCRIPTION_OF_GENES_INVOLVED_IN_G1_CELL_CYCLE_ARREST. (**G**) REACTOME_TP53_REGULATES_TRANSCRIPTION_OF_CELL_CYCLE_GENES. (**H**) REACTOME_DISEASES_OF_MITOTIC_CELL_CYCLE. (**I**) REACTOME_TP53_REGULATES_TRANSCRIPTION_OF_GENES_INVOLVED_IN_G2_CELL_CYCLE_ARREST.

When using h.all.v2022.1.Hs.symbols.gmt (Hallmarks) as the reference gene set, we identified 434 functional pathways under the condition of FDR (*q*-value) < 0.25 and *p*.adjust < 0.05. Interestingly, among these pathways, nine were associated with the cell cycle, namely REACTOME_CELL_CYCLE_CHECKPOINTS (NES = 2.733), KEGG_CELL_CYCLE (NES = 2.630), WP_CELL_CYCLE (NES = 2.623), WP_G1_TO_S_CELL_CYCLE_CONTROL (NES = 2.521), REACTOME_APC_C_MEDIATED_DEGRADATION_OF_CELL_CYCLE_PROTEINS (NES = 2.507), REACTOME_TP53_REGULATES_TRANSCRIPTION_OF_GENES_INVOLVED_IN_G1_CELL_CYCLE_ARREST (NES = 2.044), REACTOME_TP53_REGULATES_TRANSCRIPTION_OF_CELL_CYCLE_GENES (NES = 2.013), REACTOME_DISEASES_OF_MITOTIC_CELL_CYCLE (NES = 1.981), and REACTOME_TP53_REGULATES_TRANSCRIPTION_OF_GENES_INVOLVED_IN_G2_CELL_CYCLE_ARREST (NES = 1.800) (Figure 2).

### The impact of knocking down SPC25 on LUAD

**Figure 3:**
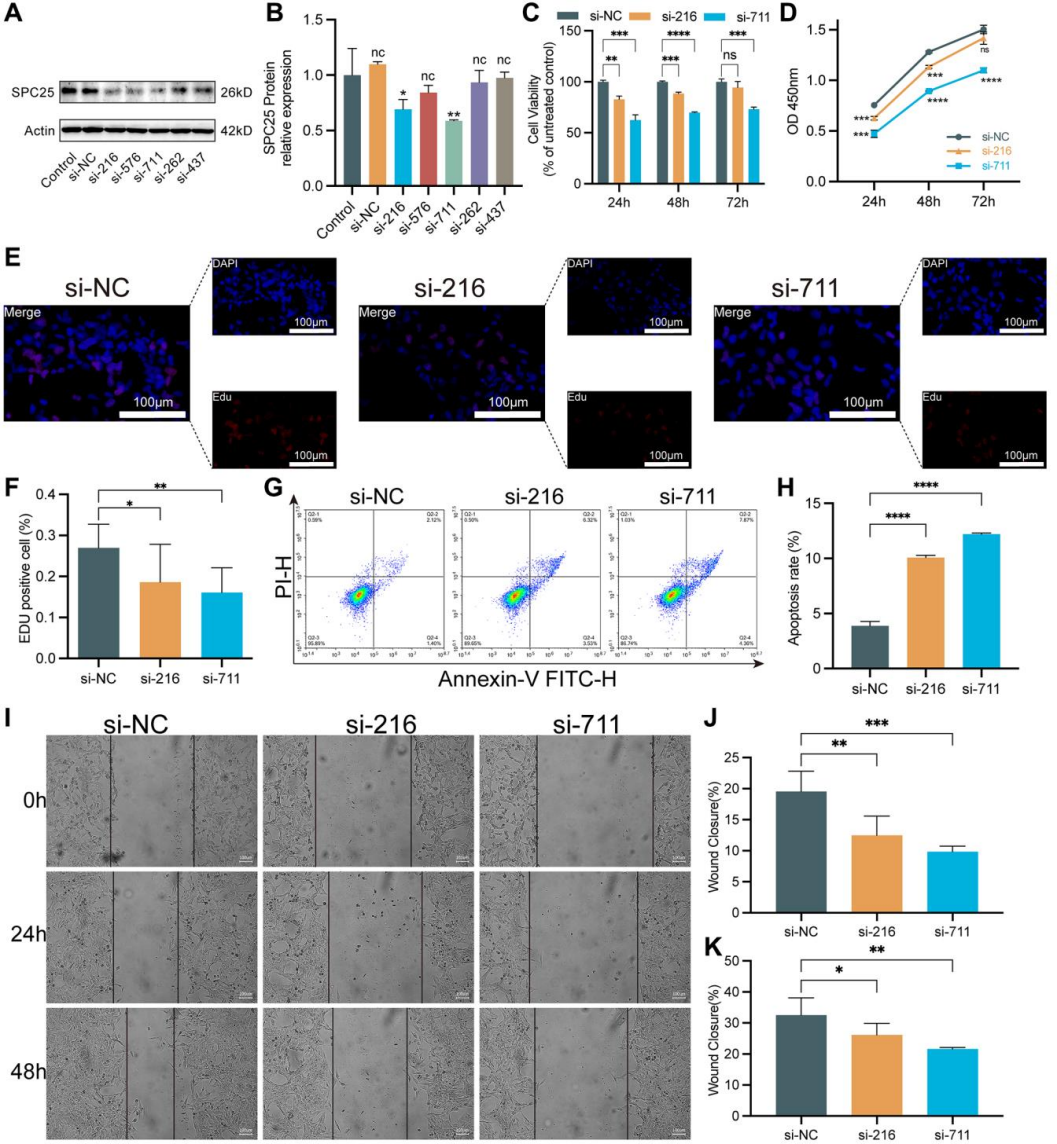
**The impact of knocking down SPC25 on LUAD.** (**A**, **B**) An analysis of Western blotting confirmed the success of SPC25-siRNAs transfection into H1975 cells and their ability to interfere with gene expression. (**C**, **D**) CCK-8 assay evaluated tumor cell proliferation activity. (**E**, **F**) EdU staining of H1975 cells and EdU-positive cell proportion. (**G**, **H**) The effect of SPC25 knockdown on apoptosis of the H1975 cells as detected by flow cytometry. (**I**–**K**) Representative images and quantitative analysis of wound healing measurements in LUAD cells. (^***^p < 0.001; ^**^p < 0.01; ^*^p < 0.05; Abbreviation: ns: not significant).

In order to evaluate the effect of SPC25 knockdown on LUAD cells, this study constructed five different sequences of siRNA to interfere with the expression of SPC25 gene. Western blot analysis confirmed the interference effects of these five siRNAs. The results showed a significant decrease in SPC25 expression after transfection with SPC25 siRNA. Among them, the interference effects of si-216 and si-711 groups were the most pronounced (Figure 3A, 3B, *P* < 0.05). Therefore, si-216 and si-711 were selected for subsequent experiments.

To evaluate the viability of LUAD cells, CCK-8 and EdU proliferation assays were utilized. The CCK-8 experiment results showed that the cell viability in the two siRNA groups was significantly lower than the control group (Figure 3C, 3D, *P* < 0.05). Similarly, in the EdU experiment, the cell viability in the two siRNA groups was significantly lower than compared to the control group (Figure 3E, 3F, *P* < 0.05). In addition, the flow cytometry results showed a significant increase in the number of apoptotic cells in the two siRNA groups, compared to the control group (Figure 3G, 3H, *P* < 0.05). Furthermore, wound healing measurement experiments were conducted, and the results demonstrated that knocking down the expression of SPC25 significantly inhibited the rate of wound healing measurement (Figure 3I–3K).

### Cell cycle regulation by SPC25 in LUAD

**Figure 4:**
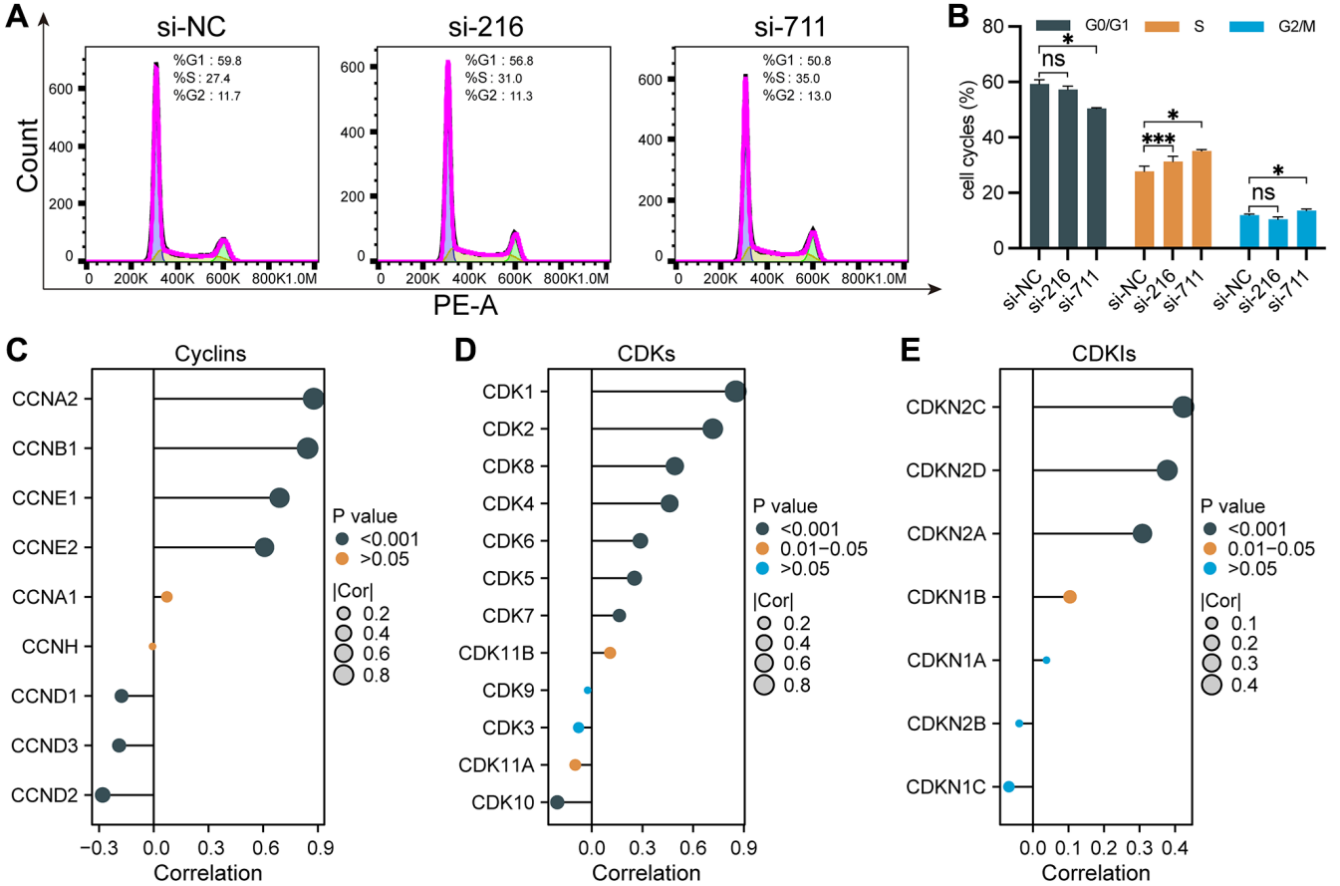
**Cell cycle regulation by SPC25 in LUAD.** (**A**, **B**) Flow cytometry analysis revealed that compared to the control group, the experimental group exhibited an increase in the percentage of cells in the S and G2/M phases, accompanied by a decrease in the percentage of cells in the G0/G1 phase, leading to cell cycle arrest at the G2/M and S phases. (**C**–**E**) We utilized lollipop plots to illustrate the correlations between SPC25 and key genes including cyclins, CDKs, and CDKIs in the TCGA LUAD dataset. (^***^p < 0.001; ^**^p < 0.01; ^*^p < 0.05; Abbreviation: ns: not significant).

As GSEA results suggested that SPC25 was positively associated with cell cycle-related pathways, we then further determined the effects of silencing SPC25 on LUAD cell cycle progression through flow cytometry. Cell cycle analysis revealed that downregulation of SPC25 resulted in cell cycle arrest in G2/M and S phases by increasing the percentage of cells in S phase and G2/M phase and decreased the percentage of cells in G0/G1 phase (Figure 4A, 4B).

Cyclins, CDKs, and CDKIs play important regulatory roles in the process of tumor occurrence and development. Dysregulated cell cycle regulation is a common characteristic of many tumors. Aberrantly expressed or functionally altered cyclins and CDKs can lead to abnormal cell cycle progression, thereby promoting tumor development. In contrast, CDKIs are generally considered to be important molecules that inhibit cell proliferation and tumor development. The correlation between SPC25 expression and these markers including 9 Cyclins, 12 CDKs and 7 CDKNs were analyzed using TCGA-LUAD data (Figure 4C–4E). The results of lollipop charts showed SPC25 was strongly correlated with most markers. As a result of the above findings, by regulating the cell cycle progression, SPC25 has the potential to affect LUAD malignant biological behaviors, such as proliferation and migration.

### SPC25 promoted glycolysis of LUAD cells

**Figure 5:**
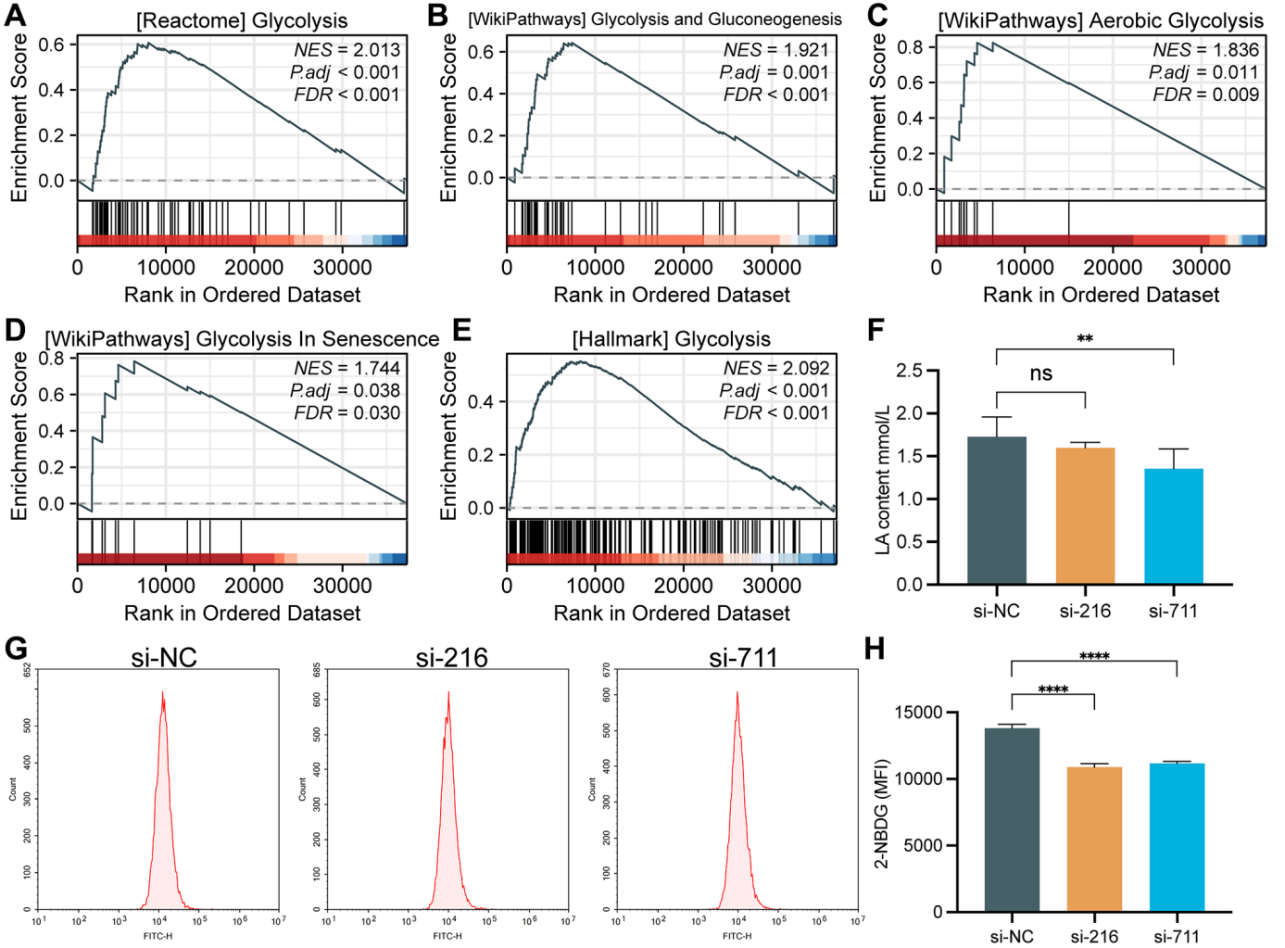
**GSEA analysis and glycolytic capacity analysis.** (**A**) REACTOME_GLYCOLYSIS. (**B**) WP_GLYCOLYSIS_AND_GLUCONEOGENESIS. (**C**) WP_AEROBIC_GLYCOLYSIS. (**D**) WP_GLYCOLYSIS_IN_SENESCENCE. (**E**) HALLMARK_GLYCOLYSIS. (**F**) Interference with SPC25 expression can significantly reduce the lactate production in LUAD cells. (**G**, **H**) Inhibition of SPC25 expression can significantly reduce the uptake of 2-NBDG by LUAD cells. MFI, mean fluorescence intensity. (^***^p < 0.001; ^**^p < 0.01; ^*^p < 0.05; Abbreviation: ns: not significant).

As shown in Figure 5A–5E, some glycolysis related pathways were activated in the high SPC25 expression group, including REACTOME_ GLYCOLYSIS, WP_GLYCOLYSIS_ AND_ GLUCONEOGENESIS, WP_AEROBIC_GLYCOLYSIS, WP_GLYCOLYSIS_IN_SENESCENCE and HALLMARK_GLYCOLYSIS. Subsequently, we measured the lactate generation and 2-NBDG glucose uptake. The results of these assays showed that the lactate generation and glucose utilization of SPC25-knockdown cells was lower than wild-type SPC25 cells (Figure 5F–5H).

**Figure 6:**
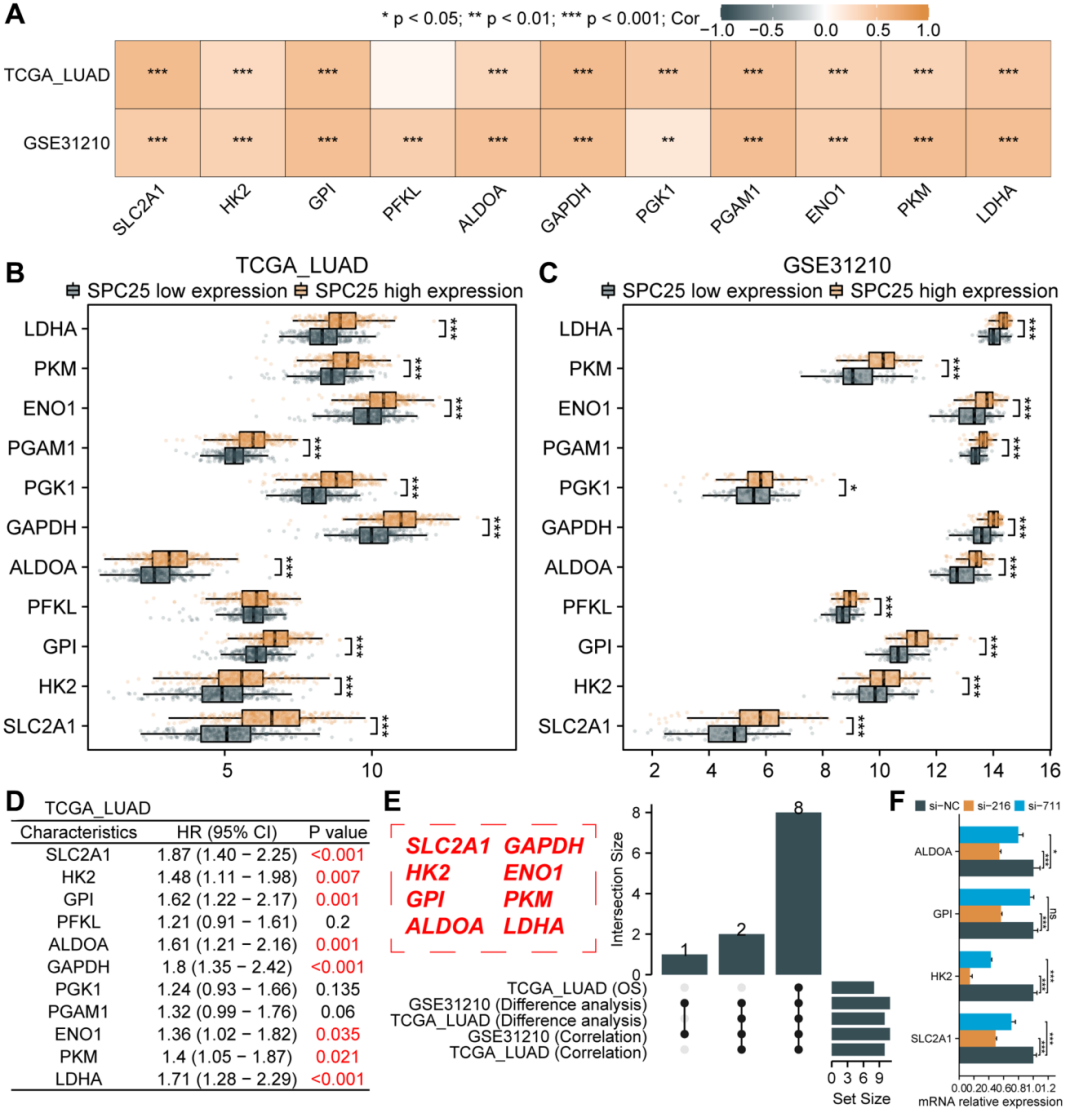
**Association between SPC25 and glycolytic-related genes.** (**A**) The correlation between SPC25 expression and 11 glycolysis-related genes was analyzed in the TCGA LUAD dataset and GSE31210 dataset. (**B**, **C**) The differential expression of 11 glycolysis-related genes between the high and low SPC25 expression groups was analyzed in the TCGA LUAD dataset and GSE31210 dataset. (**D**) Prognostic analysis of 11 glycolysis-related genes was conducted in the TCGA LUAD dataset. (**E**) The UpSet plot displays the intersection of genes with statistically significant differences in the aforementioned analyses. (**F**) Differences in the expression of 4 glycolysis-related genes between the experimental group and the control group in siRNA transfection experiments. (^***^p < 0.001; ^**^p < 0.01; ^*^p < 0.05; Abbreviation: ns: not significant).

In order to further investigate the impact of interfering with SPC25 expression on the glycolysis of LUAD cells, an analysis of glycolysis-related genes was conducted. Analysis of the TCGA LUAD dataset revealed a strong positive correlation between SPC25 expression and several glycolysis-related genes, including SLC2A1, HK2, GPI, ALDOA, GAPDH, PGK1, PGAM1, ENO1, PKM, and LDHA (Figure 6A, *P* < 0.05). Additionally, analysis of the GSE31210 dataset showed a strong positive correlation between SPC25 expression and 11 glycolysis-related genes (Figure 6A, *P* < 0.05). Furthermore, analysis of the TCGA LUAD dataset demonstrated significantly higher expression levels of SLC2A1, HK2, GPI, ALDOA, GAPDH, PGK1, PGAM1, ENO1, PKM, and LDHA in the SPC25 high-expression group compared to the low-expression group (Figure 6B, *P* < 0.05). Similarly, analysis of the GSE31210 dataset revealed higher expression levels of the 11 glycolysis-related genes in the SPC25 high-expression group compared to the SPC25 low-expression group (Figure 6C, *P* < 0.05). Further prognostic analysis identified eight genes (SLC2A1, HK2, GPI, ALDOA, GAPDH, ENO1, PKM, and LDHA) with high-expression in the TCGA LUAD dataset, which were associated with poorer survival outcomes (Figure 6D, *P* < 0.05). The UpSet plot illustrates the intersections of statistically significant genes in the above-mentioned analysis (Figure 6E, *P* < 0.05). In addition, we selected four glycolysis-related genes for cell experiments, and the results showed that compared with the control group, the expression of SLC2A1, HK2 and ALDOA genes in the experimental group was significantly down-regulated, while the expression of GPI gene was only significantly decreased in the si-216 group (Figure 6F, *P* < 0.05). These findings suggest that SPC25 may potentially regulate tumor cell glycolysis through the modulation of glycolysis-related genes, providing new insights and directions for future research in the field of tumor metabolism.

### Association between SPC25 and ferroptosis-related genes

**Figure 7:**
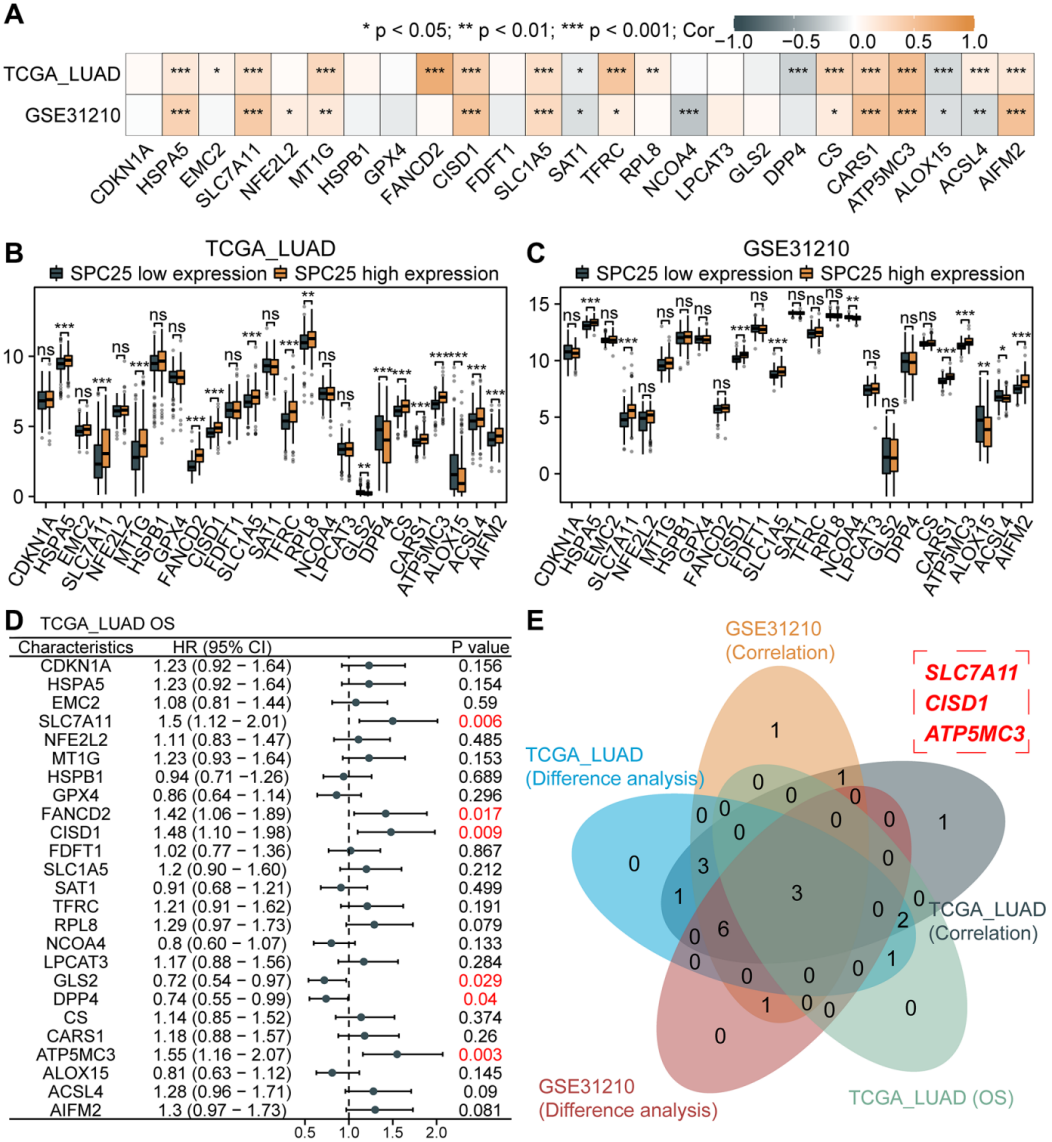
**Association between SPC25 and ferroptosis-related genes.** (**A**) The correlation between SPC25 expression and 25 ferroptosis-related genes was analyzed in the TCGA LUAD dataset and GSE31210 dataset. (**B**, **C**) The differential expression of 25 ferroptosis-related genes between the high and low SPC25 expression groups was analyzed in the TCGA LUAD dataset and GSE31210 dataset. (**D**) Prognostic analysis of 25 ferroptosis-related genes was conducted in the TCGA LUAD dataset. (**E**) The Veen plot displays the intersection of genes with statistically significant differences in the aforementioned analyses. (^***^p < 0.001; ^**^p < 0.01; ^*^p < 0.05; Abbreviation: ns: not significant).

Ferroptosis represents a novel form of cell death, holding significant relevance and necessity within the field of cellular biology. Investigating the potential association between SPC25 and ferroptosis will aid in identifying novel therapeutic targets and strategies, facilitating the development of more effective treatment approaches. We first analyzed the correlation between SPC25 expression and 25 ferroptosis-related genes in the TCGA LUAD dataset and GSE31210 dataset. The results showed that the expression of HSPA5, SLC7A11, MT1G, CISD1, SLC1A5, TFRC, CS, CARS1, ATP5MC3, and AIFM2 was significantly positively correlated with SPC25 in both datasets, while the expression of SAT1 and ALOX15 was significantly negatively correlated with SPC25 (Figure 7A, *P* < 0.05). Only one gene, ACSL4, showed opposite results in the analysis of both datasets. In addition, analysis of the TCGA LUAD dataset revealed that in the SPC25 high-expression group, the expression of HSPA5, SLC7A11, MT1G, FANCD2, CISD1, SLC1A5, TFRC, RPL8, CS, CARS1, ATP5MC3, ACSL4, and AIFM2 was significantly higher than in the low-expression group, while the expression of GLS2, DPP4, and ALOX15 was significantly lower than in the low-expression group (Figure 7B, *P* < 0.05). Analysis of the GSE31210 dataset showed that compared to the SPC25 low-expression group, the expression levels of HSPA5, SLC7A11, CISD1, SLC1A5, CARS1, ATP5MC3, and AIFM2 were higher in the SPC25 high-expression group, while the expression levels of NCOA4, ALOX15, and ACSL4 were lower in the SPC25 high-expression group (Figure 7C, *P* < 0.05). Furthermore, prognosis analysis revealed that high expression of SLC7A11, FANCD2, CISD1, and ATP5MC3 in the TCGA LUAD dataset indicated poorer survival time, while high expression of GLS2 and DPP4 indicated better survival time (Figure 7D, *P* < 0.05). Venn diagram displayed the intersection of genes with statistically significant differences in the above-mentioned analysis. These results suggest that SPC25 may potentially regulate the expression of ferroptosis-related genes SLC7A11, CISD1, and ATP5MC3, thereby influencing the iron death process in tumor cells (Figure 7E).

### Prediction and construction of the ceRNA network related to SPC25 in LUAD

**Figure 8:**
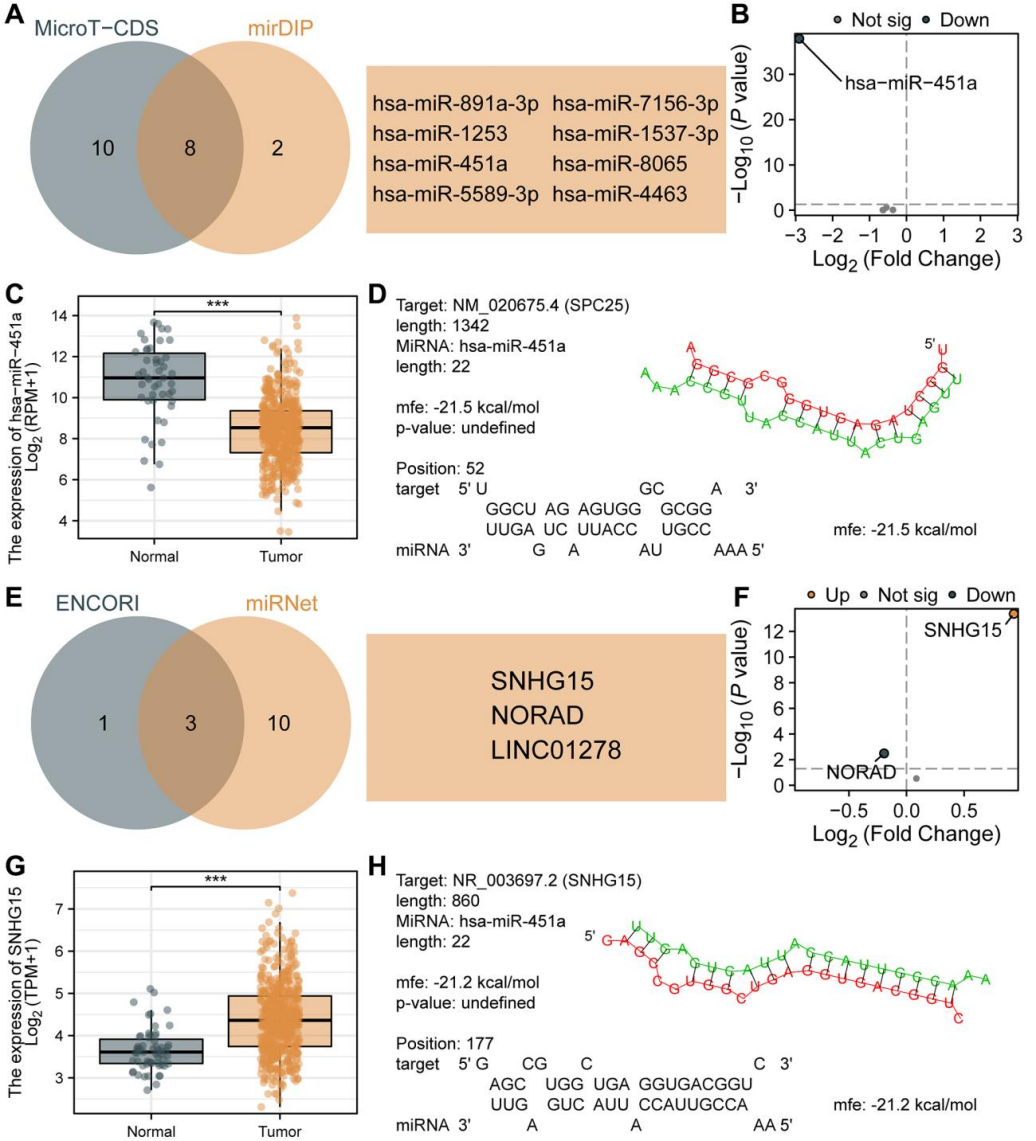
**Prediction and construction of the ceRNA network related to SPC25 in LUAD.** (**A**) The results of the analysis of microT-CDS and mirDIP databases are presented in a Venn diagram. (**B**) Differential expression analysis of 8 miRNAs in the TCGA LUAD dataset. (**C**) The expression of hsa-miR-451a in tumor samples from the TCGA LUAD dataset was significantly lower than in normal samples. (**D**) Potential binding sites between SPC25 and hsa-miR-451a were predicted using the RNAHybrid online tool. (**E**) The results of the analysis of miRNet and ENCORI databases are presented in a Venn diagram. (**F**) Differential expression analysis of 3 lncRNAs in the TCGA LUAD dataset. (**G**) The expression of SNHG15 in tumor samples from the TCGA LUAD dataset was significantly higher than in normal samples. (**H**) Potential binding sites between hsa-miR-451a and SNHG15 were predicted using the RNAHybrid online tool. (^***^p < 0.001; ^**^p < 0.01; ^*^p < 0.05; Abbreviation: ns: not significant).

Studies have shown that ceRNA is a functionally rich non-coding RNA that plays an important role in biological processes and has attracted widespread attention in the study of its functions and mechanisms in tumors. In this study, we predicted and constructed the ceRNA network related to SPC25 in LUAD. Firstly, using microT-CDS and mirDIP databases, we predicted 18 and 10 miRNAs targeting SPC25, respectively. A Venn diagram was used to select 8 miRNAs (Figure 8A), namely hsa-miR-891a-3p, hsa-miR-1253, hsa-miR-451a, hsa-miR-5589-3p, hsa-miR-7156-3p, hsa-miR-1537-3p, hsa-miR-8065, and hsa-miR-4463. Figure 8B displayed the differential expression of these 8 miRNAs in the TCGA LUAD dataset, with only hsa-miR-451a showing statistical significance. Furthermore, hsa-miR-451a exhibited significantly lower expression levels in the tumor group compared to the normal group (Figure 8C), which is consistent with the ceRNA theory. Therefore, we selected hsa-miR-451a as the targeted miRNA of SPC25, and further analyzed the potential binding sites between SPC25 and hsa-miR-451a using the RNAHybrid online tool (Figure 8D). Subsequently, using the miRNet and ENCORI databases, we predicted 13 and 4 lncRNAs that interacted with hsa-miR-451a, respectively. A Venn diagram was used to select 3 lncRNAs (Figure 8E), namely SNHG15, NORAD, and LINC01278. Figure 8F illustrates the differential expression of the above-mentioned three lncRNAs in the TCGA LUAD dataset. The analyses of SNHG15 and NORAD showed statistical significance. Moreover, the expression level of SNHG15 was significantly higher in the tumor group compared to the normal group, whereas the expression level of NORAD was significantly lower in the tumor group than in the normal group (Figure 8G). Consequently, based on the ceRNA theory, we selected SNHG15 as the targeted lncRNA for hsa-miR-451a. Furthermore, we conducted analysis using the RNAHybrid online tool to identify potential binding sites between SNHG15 and hsa-miR-451a (Figure 8H). These data suggest that the SNHG15/hsa-miR-451a/SPC25 axis may be a potential ceRNA network in LUAD, and could be closely related to tumor occurrence, development, as well as prognosis.

## DISCUSSION

LUAD, as a highly malignant and heterogeneous disease with variable prognosis, leads to nearly two million deaths annually across the world [40]. Despite clinical treatment for LUAD patients has advanced in recent decades, their 5-year survival rate remains relatively low [41–44]. Therefore, it is imperative to develop a profound understanding of etiological factors and more efficient therapeutic approaches to understand the intricate progression within LUAD. There are several facets to the significance of our present findings: Firstly, it has been observed that SPC25 is upregulated in various tumors, which is associated with unfavorable staging and prognosis in LUAD. Secondly, our study demonstrates that inhibition of SPC25 reduces the growth of LUAD cells and induces alterations in the cell division process, accompanied by downregulation of glycolysis-related genes. Finally, by analyzing various databases, it was found that the expression of SPC25 was significantly correlated with ferroptosis-related genes, and it was also found that SPC25 may be involved in the regulation of the ceRNA network of LUAD.

SPC25 is an important cell cycle protein involved in chromosome separation and spindle dynamics regulation during mitosis. It is one of the indispensable components of cell division and a key process related to tumorigenesis [45–47]. To date, research on SPC25 has mainly focused on its function during mitosis, and it has been established that its dysregulation can lead to abnormal cell division. However, the data available on the association between SPC25 and carcinogenesis are still very limited. Recent studies have reported the overexpression of SPC25 in various tumors, including head and neck cancer, breast cancer, prostate cancer, lung cancer, hepatocellular carcinoma (HCC), colorectal cancer, and gastric cancer [48–54]. Furthermore, the overexpression of SPC25 has been associated with poor prognosis in patients with head and neck cancer, breast cancer, prostate cancer, and HCC [48, 49, 51, 53, 54]. Additionally, it has been found that reducing the expression of SPC25 can significantly inhibit the progression of head and neck cancer, prostate cancer, and liver cell carcinoma [48, 50, 53, 54]. Several studies have also reported significantly increased expression of SPC25 in LUAD tumors. High expression of SPC25 is associated with low OS [11, 55]. Decreased SPC25 expression can significantly inhibit the proliferation of LUAD cells [10, 11]. However, the mechanism behind this process remains unclear, and further exploration of the biological pathways associated with LUAD is lacking, which is the focus of this study.

In this study, we verified the high expression of SPC25 in LUAD through various database analyses, and its high expression was associated with poor prognosis of LUAD. We also found that the expression level of SPC25 was significantly correlated with pathological staging, which is consistent with earlier studies [10, 11, 55]. We also found that interfering with the expression of SPC25 can significantly inhibit the proliferation and migration of LUAD cells, and promote the apoptosis of tumor cells. Compared to previous studies, this study used more detection methods. It has been reported that SPC25 is an important cell cycle protein, and we found that the expression level of SPC25 can affect the cell cycle progression of LUAD cells through GSEA analysis and cell cycle experiments. Further correlation analysis found that the expression of SPC25 was significantly correlated with most genes related to cyclins, CDKs and CDKIs, which further confirmed the important role of SPC25 in the cell cycle.

In this study, we have used GSEA analysis to discover the correlation between high expression of the SPC25 gene and multiple glycolytic pathways. Furthermore, we have validated the crucial role of SPC25 in glucose uptake and lactate production in tumor cells through cellular experiments. These findings provide important clues for a better understanding of the regulatory mechanisms of tumor cell energy metabolism. Glycolysis, as a major energy supply pathway, plays a critical role in tumor cells [25, 56]. In our further experimental investigations, we have downregulated the expression of SPC25 and observed a significant inhibition of glucose uptake and lactate production in tumor cells. This suggests that SPC25 plays a pivotal regulatory role in tumor cell energy metabolism. Through the analysis of multiple datasets, we have identified potential connections between SPC25 and glycolysis-related genes, including SLC2A1, HK2, GPI, ALDOA, GAPDH, PGK1, PGAM1, ENO1, PKM, and LDHA. These genes are key regulatory factors in the glycolytic process and their abnormal expression is closely associated with tumor occurrence and development [25, 26]. In addition, prognosis analysis results reveal a correlation between high expression of SLC2A1, HK2, GPI, ALDOA, GAPDH, ENO1, PKM, and LDHA in LUAD and poor prognosis, further highlighting the significance of these genes in tumor occurrence and development. At the same time, our cell experiments found that the expression of SLC2A1, HK2, GPI and ALDOA genes in the experimental group was significantly down-regulated compared with the control group. Compared to existing research, our study provides in-depth research on the role of SPC25 in tumor cell energy metabolism and uncovers potential connections with glycolysis-related genes. This offers a new perspective on the precise regulatory mechanisms of tumor cell energy metabolism and provides new targets for developing tumor treatment strategies targeting the glycolysis pathway. However, further research is still needed to explore the interaction network between SPC25 and glycolysis-related genes, as well as the specific regulatory mechanisms of these genes in tumor occurrence and development.

Recent studies have shown that abnormalities in ferroptosis are commonly present in various cancers, but there have been no reports on the relationship between SPC25 and ferroptosis. In this study, the TCGA-LUAD and GSE31210 datasets were utilized, revealing the potential links between SPC25 and ferroptosis-associated genes, namely SLC7A11, CISD1, and ATP5MC3. Furthermore, previous research has demonstrated that METTL3-mediated m6A modification can stabilize SLC7A11 mRNA and promote its translation, thus facilitating proliferation and inhibiting iron-induced cell death in LUAD cells [57]. Another study found that SLC7A11, CISD1, and ATP5MC3 are overexpressed in LUAD samples, and knockdown of CISD1 significantly suppresses proliferation and migration in LUAD [58]. These findings suggest that SPC25 may serve as a potential therapeutic target in LUAD. By modulating the expression of SPC25, it may be possible to intervene in the function of SLC7A11, CISD1, and ATP5MC3, thereby affecting the proliferation and migration abilities of LUAD cells. This discovery provides new clues for further investigating the relationship between SPC25 and ferroptosis.

In recent years, more and more studies have shown that ceRNA plays an important role in the normal physiological process of cells and the occurrence and development of tumors [19, 20]. However, there is no relevant report on the ceRNA network study of the SPC25 gene. In this study, miRNAs capable of targeting SPC25 were confirmed by preliminary screening using various databases, and differential analysis and target prediction were performed to determine that SPC25 is a potential target of hsa-miR-451a. In further research, we predicted a lncRNA (SNHG15). According to the ceRNA theory, we speculated that SNHG15 may target hsa-miR-451a, and finally constructed a ceRNA network, namely SNHG15/hsa-miR-451a/SPC25. Huang et al. found that SNHG15 can alter the chemotherapy resistance of LUAD cells to gefitinib by regulating miR-451/MDR-1 [59]. This finding is consistent with our findings, which further prove that SNHG15 has important biological functions in LUAD as an upstream lncRNA of hsa-miR-451a. To sum up, our study preliminarily constructed a ceRNA network involving SPC25 genes, which provides a strong basis for in-depth exploration of the interaction between SPC25 and other factors. However, further research is needed to verify our speculation and explore the mechanism of action and clinical application prospects of ceRNA networks in other cancers.

## CONCLUSION

In conclusion, we elucidate that increased SPC25 expression in LUAD is closely associated with poor prognosis and staging. Interfering with SPC25 expression can significantly inhibit the proliferation and migration of LUAD cells, and promote apoptosis and affect cell cycle progression. The study also found that the expression level of SPC25 is closely related to cyclins, CDKs, CDKIs, glycolysis and ferroptosis-related genes, and may promote LUAD progression through the SNHG15/hsa-miR-451a/SPC25 pathway, however, its detailed relationship remains to be determined. In conclusion, this study enhances our understanding of the molecular mechanism of LUAD and lays the foundation for future research on targeted therapies involving the SPC25 gene.

## Supplementary Materials

Supplementary Table 1
