# Supplementary material for: SPC25 as a novel therapeutic and prognostic biomarker and its association with glycolysis, ferroptosis and ceRNA in lung adenocarcinoma
Source: Aging (Albany NY). 2024 Jan 11;16(1):779–98. doi: 10.18632/aging.205418 (PMC10817414; doi:10.18632/aging.205418)
Supplement: Supplementary Table 1 [file aging-16-205418-s001.pdf]

SUPPLEMENTARY TABLE

Supplementary Table 1. The sequences for all primers in our study.

Primer name	Primer sequence (5′–3′)
β-actin F	TGGCACCCAGCACAATGAA
β-actin R	CTAAGTCATAGTCCGCCTAGAAGCA
SLC2A1 F	CACTGTCGTGTCGCTGTTTG
SLC2A1 R	CTAGCGCGATGGTCATGAGT
HK2 F	GGCAAGCAGAGGTTCGAGA
HK2 R	AAGTGTTGCAGGATGGCTCG
GPI F	AAGGGTCTGCATCACAAGATCC
GPI R	AGAGTTGGTTGGGCGATTTCC
ALDOA F	ACATCGCTCACCGCATCGT
ALDOA R	GGTAGTCTCGCCATTTGTCCC
SPC25 F	GACACCTCCTGTCAGATGGC
SPC25 R	TTTGCCTGCTGATCTGATTTTGA
